# Role of conserved *cis*-regulatory elements in the post-transcriptional regulation of the human MECP2 gene involved in autism

**DOI:** 10.1186/1479-7364-7-19

**Published:** 2013-09-16

**Authors:** Joetsaroop S Bagga, Lawrence A D’Antonio

**Affiliations:** 1 John P. Stevens High School, 855 Grove Ave., Edison, NJ 08820, USA; 2 Ramapo College of New Jersey, 505 Ramapo Valley Rd., Mahwah, NJ 07430, USA; 3 Carnegie Mellon University, 5000 Forbes Ave., Pittsburgh, PA 15213, USA

**Keywords:** G-quadruplex, Post-transcriptional regulation, MECP2, MicroRNAs, AU-rich elements, Autism

## Abstract

**Background:**

The MECP2 gene codes for methyl CpG binding protein 2 which regulates activities of other genes in the early development of the brain. Mutations in this gene have been associated with Rett syndrome, a form of autism. The purpose of this study was to investigate the role of evolutionarily conserved *cis-*elements in regulating the post-transcriptional expression of the MECP2 gene and to explore their possible correlations with a mutation that is known to cause mental retardation.

**Results:**

A bioinformatics approach was used to map evolutionarily conserved *cis*-regulatory elements in the transcribed regions of the human MECP2 gene and its mammalian orthologs. *Cis-*regulatory motifs including G-quadruplexes, microRNA target sites, and AU-rich elements have gained significant importance because of their role in key biological processes and as therapeutic targets. We discovered in the 5′-UTR (untranslated region) of MECP2 mRNA a highly conserved G-quadruplex which overlapped a known deletion in Rett syndrome patients with decreased levels of MeCP2 protein. We believe that this 5′-UTR G-quadruplex could be involved in regulating MECP2 translation. We mapped additional evolutionarily conserved G-quadruplexes, microRNA target sites, and AU-rich elements in the key sections of both untranslated regions. Our studies suggest the regulation of translation, mRNA turnover, and development-related alternative MECP2 polyadenylation, putatively involving interactions of conserved *cis-*regulatory elements with their respective *trans* factors and complex interactions among the *trans* factors themselves. We discovered highly conserved G-quadruplex motifs that were more prevalent near alternative splice sites as compared to the constitutive sites of the MECP2 gene. We also identified a pair of overlapping G-quadruplexes at an alternative 5′ splice site that could potentially regulate alternative splicing in a negative as well as a positive way in the MECP2 pre-mRNAs.

**Conclusions:**

A Rett syndrome mutation with decreased protein expression was found to be associated with a conserved G-quadruplex. Our studies suggest that MECP2 post-transcriptional gene expression could be regulated by several evolutionarily conserved *cis-*elements like G-quadruplex motifs, microRNA target sites, and AU-rich elements. This phylogenetic analysis has provided some interesting and valuable insights into the regulation of the MECP2 gene involved in autism.

## Background

The methyl CpG binding protein 2 gene codes for the protein MeCP2, which is essential for normal brain development [1]. This protein is responsible for regulated transcription of neuron-specific genes and is vital for connecting nerve cells, where cell–cell communication takes place. Mutations in the MECP2 gene can cause a form of autism called Rett syndrome. Victims of this syndrome are typically females between the ages of 6 and 18 months. Additionally, Rett syndrome patients experience a loss of acquired skills, impaired speech, and abnormal stereotypical movements. In some cases, young patients have experienced frequent seizures and mental retardation [2]. Rett syndrome is in fact one of the most common causes of mental retardation in females.

Several types of mutations have been mapped to the MECP2 gene from affected patients [3,4]. Many of the mutations affect the coding region and either result in a MeCP2 protein with altered function or a non-functional protein. Mutations that lead to altered gene expression have been mapped to the 5′- and 3′-untranslated regions (UTRs) [3,5,6]. Several mutations in the genomic MECP2 sequence lead to altered splicing of the gene [3].

C*is*-regulatory motifs located in the untranslated regions and in the vicinity of splice junctions are known to interact with RNA binding proteins for regulating post-transcriptional gene expression. Studying *cis*-element regulation of MECP2 gene expression can help provide better insights into the molecular mechanism of MECP2 regulation and deeper understanding of the genetic disorders caused by alteration of its expression.

Guanine-rich sequences can form highly stable structures. Instead of the Watson and Crick DNA duplex, four consecutive tetrads of G-rich sequences in a nucleic acid can form G-quadruplexes [7]. The G-quadruplexes are known to have important roles in biological processes and human disease and as therapeutic targets [8-11]. These structures have been found in telomeres, promoter regions, and other biologically important regions in the DNA influencing DNA replication, transcription, and epigenetic mechanisms [12,13]. Computationally predicted G-quadruplex structures have been reported in the MECP2 gene [14]. However, the biological role of these motifs in the MECP2 DNA remains to be determined. Recently, it became possible to quantitatively visualize the formation of genomic G-quadruplexes in living mammalian cells [15]. RNA G-quadruplexes are more likely to be formed *in vivo*[16] and are more stable than the DNA G-quadruplexes [17]. There is ample evidence for *cis-*regulatory roles of G-quadruplexes in the post-transcriptional gene expression [18]. RNA G-quadruplexes located in the 5′-UTR have been known to be involved in regulated translational initiation [19,20] as well as translation repression [21-23]. G-quadruplex motifs found in the translated regions have been shown to affect folding and proteolysis of hERα protein [24]. G-rich sequences in the 3′-UTR have been shown to influence polyadenylation [25], RNA turnover [26], and subcellular mRNA localization [27]. A 3′-UTR polymorphism that affects G-quadruplex structure has been shown to modulate gene expression of the KiSS1 mRNA [28]. There is evidence for direct G-quadruplex role in regulated alternative splicing of fragile X mental retardation 1 (FMR1) transcripts [29] and of beta-site amyloid precursor protein (APP) cleaving enzyme 1 (BACE1) involved in Alzheimer disease [30].

Development of bioinformatics techniques has made it possible to study the prevalence and distribution of G-quadruplex forming sequence motifs at genomic levels [31-34]. Consequently, there has been a tremendous increase in published literature and reviews on this subject [34-36]. Large scale computational studies have identified an association of G-quadruplex forming sequences in both 5′- as well as 3′-UTRs [37]. However, computational predictions have difficulty in distinguishing between a G-quadruplex sequence motif which occurs by chance and the one that forms a structure with a biological role in the cell.

In this study, we have used a bioinformatics approach to map evolutionarily conserved G-quadruplex motifs, microRNA target sites, and AU-rich elements (AREs) in the transcribed regions of the human MECP2 gene and its mammalian orthologs. Identifying evolutionarily conserved motifs helps validate computational predictions, improving accuracy, and providing evidence for their biological relevance. The goal of this project was to study the role of conserved *cis*-regulatory motifs in regulating the post-transcriptional expression of the MECP2 gene and explore their possible correlations with a mutation that is known to cause mental retardation.

The translation and destabilization of large number of eukaryotic mRNAs are known to be regulated via microRNA-mediated pathways, which have received significant attention [38]. MeCP2 protein expression has been shown to be influenced by microRNA targeting [39]. Similarly, AU-rich elements in the 3′-UTRs of developmentally expressed mRNAs have been associated with regulated stability [40]. Therefore, in addition to the G-quadruplexes, the roles of microRNA targeting and AREs as post-transcriptional regulators and their interrelationships were also investigated in this project.

## Results and discussion

A total of four MECP2 mammalian orthologs, *Homo sapiens, Canis lupus familiaris*, *Mus musculus,* and *Rattus norvegicus* were chosen for the current studies (Table 1). Although the MeCP2 protein orthologs were quite similar, the nucleotide sequence similarities among the mRNAs were relatively lower due to variation in the 5′- and 3′- untranslated regions (human, dog, and mouse MECP2 genes are known to have multiple isoforms. Orthologous isoforms with comparable exon/intron structures were chosen for sequence alignments.).

**Table 1:** A total of four MECP2 mammalian orthologs were chosen for the current studies

**MECP2 ortholog**	**Nucleotide sequence (mRNA) identity to human**	**Protein sequence identity/similarity to human**	**Protein length (amino acids)**
*Homo sapiens* [RefSeq:NM_004992.3]	100%	100%	486
*Canis lupus familiaris* [RefSeq:XM_848395.1]	92.50%	96%/97%	486
*Mus musculus* [RefSeq:NM_001081979.1]	85.00%	95%/96%	501
*Rattus norvegicus* [RefSeq:NM_022673.1]	78.20%	94%/95%	492

RNAs were aligned pairwise by semi-global method which does not penalize end-gaps. Therefore, the percentage similarity calculations do not include differences in the lengths of untranslated regions. Values in parentheses represent NCBI accession numbers of the corresponding mRNAs.

### A conserved G-quadruplex in the 5′-UTR of MECP2 orthologs

**Figure 1:**
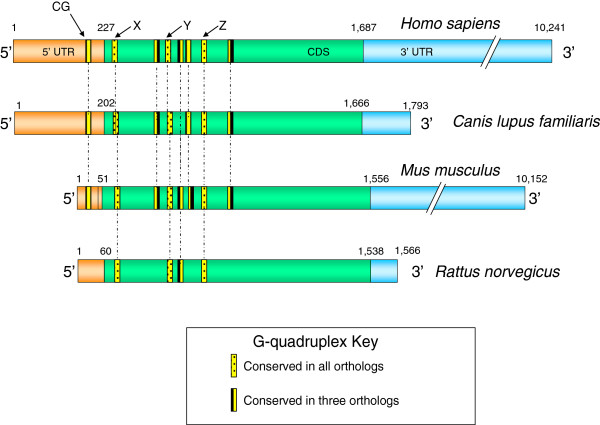
**The G-quadruplex map of MECP2 mRNA orthologs.** The 5′-UTR and CDS region G-quadruplexes are conserved in terms of their relative location to the translation start site in the MECP2 mRNA orthologs. Based on their observed conservation level, G-quadruplexes have been categorized into groups. Location conserved G-quadruplexes ‘CG’ (in the 5′-UTR), ‘X,’ ‘Y,’ and ‘Z’ (in the CDS) were subjected to further sequence analysis.

**Figure 2:**
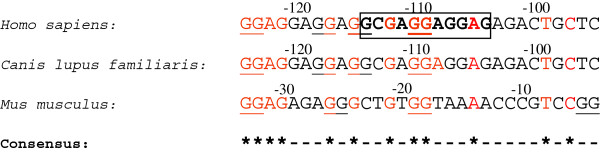
**The conserved G-quadruplex motif (CG).** This motif in the 5′-UTR of *Homo sapiens*, *Canis lupus familiaris*, and the *Mus musculus* MECP2 mRNAs maps to a known deletion in the human MECP2 gene leading to Rett syndrome. The predicted tetrad forming G-tracts of the quadruplex are underlined. This conserved G-quadruplex is likely to get disrupted due to the 11-bp deletion which is known to affect MECP2 translation in some Rett syndrome patients*.* The deletion is marked in the box with bold characters. The numbers represent upstream distances from the CDS start sites of the respective mRNAs.

A G-quadruplex highly conserved in relative location to the translation start site was discovered in the 5′-UTR of human, dog, and mouse MECP2 mRNAs (Figure 1). Existence of a conserved motif within an otherwise highly variable region signifies its functional role. This conserved G-quadruplex motif, which we named ‘CG’ , is located 110 bases upstream of the translation initiation site in the human MECP2 mRNA and is likely to play a role in the regulation of translation. There have been several reports of 5′-UTR G-quadruplexes that are involved in translation regulation. A G-quadruplex structure located in the 5′-UTR of human fibroblast growth factor 2 (FGF2) acts as an internal ribosomal entry site (IRES) for translation initiation [19]. On the other hand, formation of G-quadruplexes can also play inhibitory roles for translation of NRAS oncogene [21], Ying Yang 1 involved in tumorigenesis [41], and ADAM10 responsible for anti-amyloidogenic processing of the APP [22]. The CG G-quadruplex conserved in the 5′-UTR of human, dog, and mouse MECP2 mRNA orthologs (Figures 1 and 2) is of particular interest because it maps to a known mutation in the MECP2 gene leading to Rett syndrome [42]. An 11-bp deletion (GCGAGGAGGAG) (Figure 2) in the 5′-UTR results in the lack of MeCP2 protein in about 25% of the tested cells even though the mRNA is detectable and the coding sequence (CDS) of the mRNA is apparently intact [42].

We believe that the MECP2 5′-UTR G-quadruplex CG is in fact the translation regulatory motif which gets affected due to the 11-bp deletion in some Rett syndrome patients. Nucleotide sequence mutations and polymorphisms that destroy G-quadruplex folding or change the G-quadruplex conformation are known to affect gene expression [22,28]. Two possible mechanisms may lead to G-quadruplex-mediated regulation of translation in the MECP2 mRNA. Interaction of RNA binding proteins with the G-quadruplexes in the 5′-UTR is known to modulate translation. For example, nucleolin protein binds to G-rich sequences to positively influence protein translation [43]. We have tested several nucleolin targets [43] with the quadruplex forming G-rich sequences (QGRS) Mapper software [31] and found them to be capable of forming G-quadruplexes (data not shown). A disruption in the 5′-UTR G-quadruplex of the MECP2 mRNA could consequently lead to lower protein translation. The fragile X mental retardation protein (FMRP) is also known to regulate translation by binding to G-quadruplexes on its target mRNAs [44]. Altered function of FMRP could lead to atypical synapse development in the brain and impaired learning resulting in mental retardation [45]. Several other genes implicated in autism have been shown to form G-quadruplexes [44,46]. A change in the 5′-UTR G-quadruplex region is likely to affect FMRP binding and hence translation of MECP2 mRNA, possibly leading to genetic defects like Rett syndrome.

Alternatively, the 5′-UTR G-quadruplex may be an important component of IRES [19,20] which is responsible for translation of the Mecp2 mRNA. The 11-bp deletion in the G-quadruplex motif, and therefore disruption of IRES, may affect the translation of the Mecp2 mRNA.

### Conserved G-quadruplexes in the coding region of MECP2 orthologs

**Figure 3:**
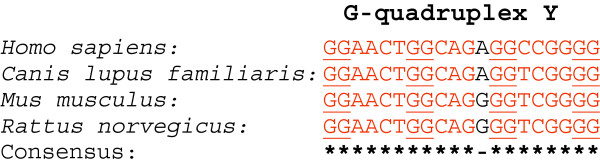
**Location-conserved G-quadruplex ‘Y’ in the CDS of MECP2 orthologs.** This motif showed a high level of sequence conservation across the four mammalian species. G-quadruplex ‘Y’ refers to the corresponding marked map position in the Figure 1 above.

We mapped several conserved G-quadruplexes within the CDS region of the MECP2 mRNA orthologs. Three G-quadruplexes (‘X’ , ‘Y’ , and ‘Z’ , Figure 1) were highly conserved within the MECP2 CDS region of all four species. The G-quadruplex ‘Y’ showed a high level of sequence conservation across the four mammalian species (Figure 3). Regardless of the modest variation in sequence conservation, all of the three CDS G-quadruplexes exhibited high conservation at a position relative to the translation start site and at the predicted structure level. G-quadruplexes within the coding regions of mRNAs are known to be involved in regulating the RNA stability [47], translation [43], and protein folding [24].

### Conserved *cis*-regulatory elements in the 3′-UTR of MECP2 orthologs

The MECP2 mRNAs analyzed in this work included two alternatively spliced isoforms each for human, dog, and mouse orthologs and one MECP2 transcript of rat. Both MECP2 isoforms of mouse and human isoform 1, each have long 3′-UTRs (>8.5 kb). Both of the dog MECP2 isoforms, isoform 2 of human MECP2 and the rat mRNA each have short 3′-UTRs (<0.5 kb). The longer MECP2 isoforms contain at least two polyadenylation signals and their corresponding cleavage/polyadenylation sites. Alternative polyadenylation in MECP2 can lead to transcript isoforms with the longer or shorter version of the 3′-UTRs [48]. The longer human isoform has been found to be in higher abundance in the fetal neuronal tissues and involved in the development of the brain while shorter transcripts are prevalent within the adult brain [48]. Long 3′-UTRs are likely to play pivotal roles in post-transcriptional regulation of MECP2 mRNA, especially during the early developmental process when gene expression needs to be tightly regulated. Therefore, this part of our project explored the capability of 3′-UTRs of MECP2 mammalian orthologs and isoforms to form evolutionarily conserved G-quadruplexes, especially in the vicinity of other conserved *cis*-regulatory elements: AREs, microRNA target sites, and alternative polyadenylation signals.

First, we studied the overall phylogenic conservation of the MECP2 gene particularly in the 3′-UTR regions. Based on sequence alignments among mammalian orthologs of MECP2 mRNAs, we found that most of the MECP2 3′-UTR sequence is highly variable. However, regions surrounding polyadenylation signals/sites showed much better conservation (data not presented). This suggests important biological roles of the conserved regions in the regulation of alternative polyadenylation involved in the developmental regulation of MECP2.

**Figure 4:**
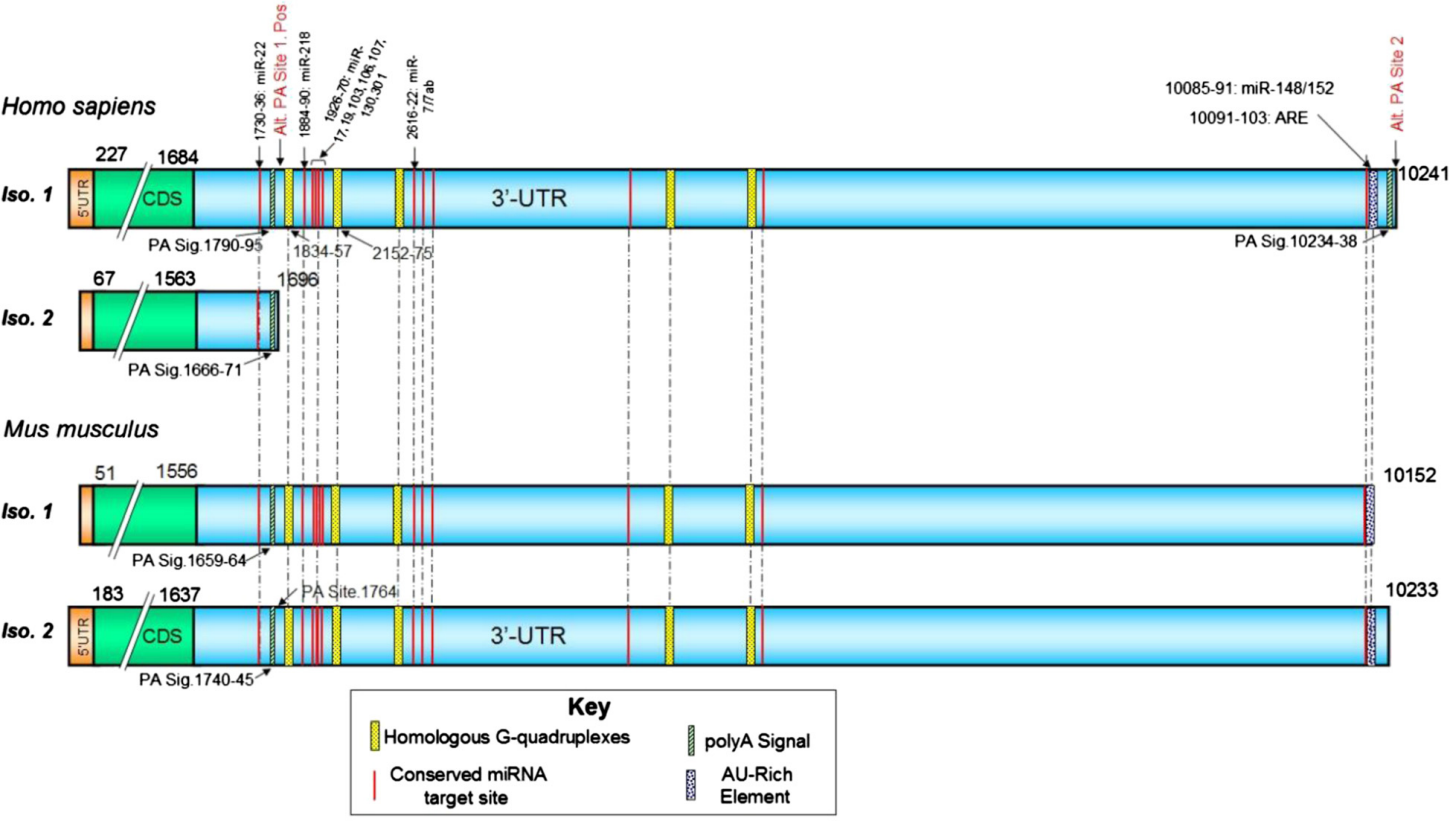
**Conserved 3′-UTR *cis-*regulatory elements map of MECP2 mRNA orthologs and isoforms.** Majority of the *cis*-regulatory elements mapped to the evolutionarily conserved regions of long MECP2 isoform 3′-UTR which is involved in early brain development. All four mammalian orthologs of MECP2 mRNAs from human, dog, mouse, and rat were analyzed. Only human and mouse mRNA alignments are displayed. Human MECP2 alignments with its dog and rat orthologs were very similar to the alignment shown. The short human MECP2 isoform 2 lacked conserved microRNA targets, ARE, or G-quadruplexes. A highly conserved G-quadruplex is present selectively near one alternative polyadenylation signal/site. Most evolutionarily conserved G-quadruplexes were preferentially associated with microRNA target sites. Evolutionarily conserved AU-rich element (ARE) and mi-R148/152 target sites were associated with the second alternative poly(A) site which results in the expression of longer isoform during the early development of the human brain.

The 3′-UTR of MECP2 is highly variable; however, the majority of the conserved *cis*-regulatory elements that we analyzed (microRNA target sites, AU-rich elements, and G-quadruplexes) mapped to evolutionarily conserved regions in the 3′-UTR of the long MECP2 isoform, which is involved in early brain development (Figure 4) (all four mammalian orthologs of MECP2 were analyzed. Only data from human and mouse isoforms is presented. Human MECP2 alignments with its dog and rat orthologs were very similar to the alignments between human and mouse orthologs). The short human MECP2 mRNA isoform 2, expressed mostly in the adult brain, lacked conserved microRNA targets, ARE, or G-quadruplexes. Our results suggest that these conserved *cis*-elements could have important regulatory roles in post-transcriptional MECP2 expression during early development stages of the brain.

**Figure 5:**
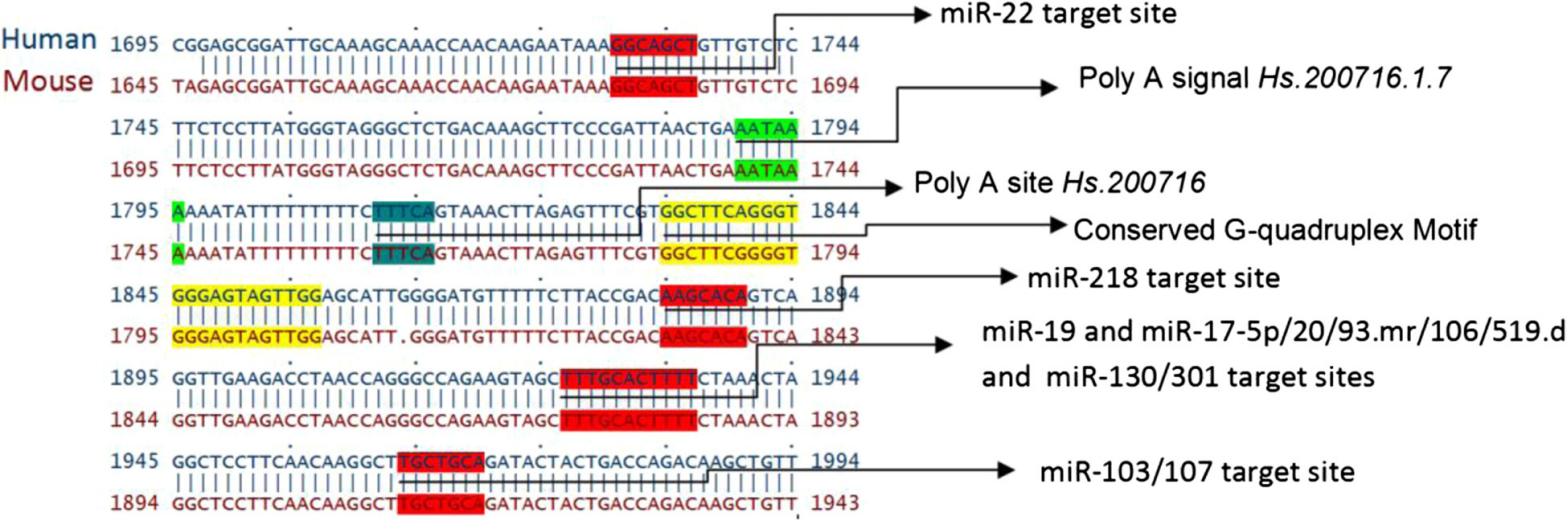
**Conserved *cis-*regulatory elements associated with alternate poly(A) site 1 of MECP2 mRNA.** A conserved G-quadruplex and several conserved microRNA target sites are associated with alternative polyadenylation site1.

**Figure 6:**
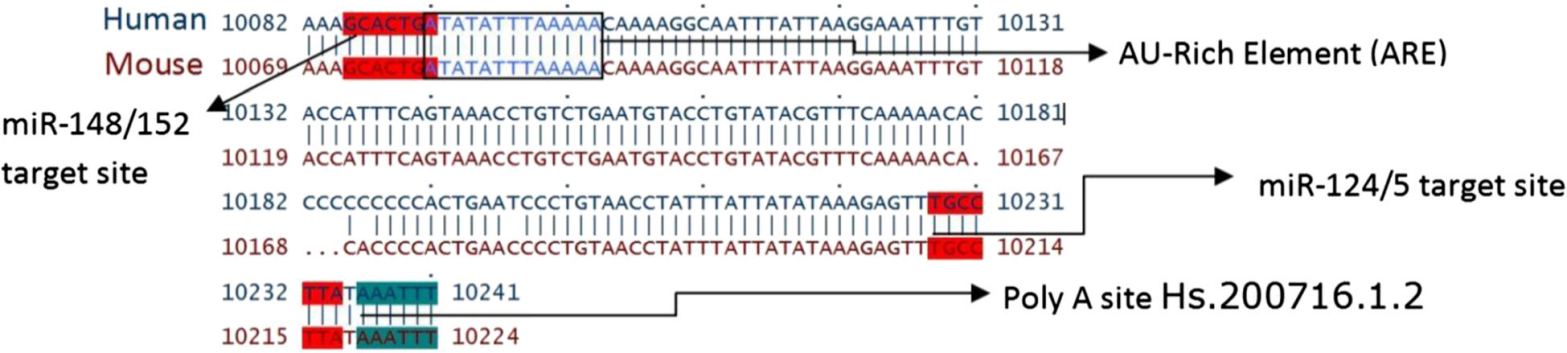
**Conserved *cis-*regulatory elements associated with alternate poly(A) site 2 of MECP2 mRNA.** Evolutionarily conserved AU-rich element (ARE) and mi-R148/152 target sites are associated with the second alternative poly(A) site which results in the expression of the longer isoform during the early development.

There is sufficient evidence to indicate a role for 3′-UTR G-quadruplex in post-transcriptional regulation of gene expression [28,43,49-51]. G-quadruplexes in the 3′-UTR are known to regulate translation [43]. Interactions between RNA binding proteins like hnRNP F/H and quadruplex forming G-rich sequences are known to regulate splicing and 3′-end processing [49-51]. In our studies, a highly conserved G-quadruplex was found to be associated with one alternative poly(A) site but not the second site (Figure 4). The conserved G-quadruplex was present 17 bases downstream of the poly(A) site 1 (Figure 5), well within the range of the cleavage/polyadenylation complex formation associated with G-quadruplex-mediated regulation of 3′-end formation [49]. Mutations of G-rich sequences in this region of MECP2 RNA have been shown to reduce polyadenylation efficiency *in vivo*[52]. We did not find any evidence of G-quadruplex forming sequences within 200 bases downstream of the alternative poly(A) site 2 responsible for the long isoform of the human MECP2 gene (Figure 6 and data not shown). These results suggest a G-quadruplex role in alternative cleavage/polyadenylation associated with brain development-specific MECP2 gene expression. The mechanism of alternative 3′-end processing regulation may involve dynamic formation or resolution of the RNA G-quadruplex near poly(A) Site 1 via specific helicases such as RHAU [53]. The role of G-quadruplexes in polyadenylation can be modulated by interactions with different proteins. For example, while binding of hnRNP H/H′ to quadruplex forming G-rich sequences can enhance polyadenylation [49,54], hnRNP F (which also has affinity for G-rich tracts) has been shown to interfere with polyadenylation [55].

Most of the evolutionarily conserved microRNA target sites were located in 3′-UTR of the long isoform; many of them are approximately 100 bp downstream of the poly(A) site 1 which is closer to the MECP2 coding region (Figure 4). The translation and destabilization of a large number of eukaryotic mRNAs, especially those under strict expression regulation, are known to be regulated via microRNA-mediated pathways [38]. Therefore, it was not surprising to discover microRNA target sites in the 3′-UTR of developmentally regulated long MECP2 isoform. MicroRNA targeting the long 3′-UTR MECP2 isoform has been previously shown to modulate MeCP2 protein levels in the developing human brain [56].

We noticed that most evolutionarily conserved G-quadruplexes were preferentially associated with conserved microRNA target sites in the 3′-UTR (Figure 4), suggesting a potential interplay between microRNAs/microRNP (microribonucleoprotein) and G-quadruplex binding proteins. G-quadruplex binding proteins like FXR1 (fragile X retardation 1, a paralog of FMRP and involved in mental retardation) are known to be part of microRNP complexes [57]. FXR1 is also involved in directing microRNAs to the ARE for regulation of translation [57]. Therefore, a regulatory role for some G-quadruplexes in 3′-UTR of MECP2 may also have to do with mRNA translation.

Evolutionarily conserved ARE and mi-R148/152 target sites were associated with the second alternative poly(A) site which results in the expression of longer isoform (Figures 5 and 6). AU-rich elements in the 3′-UTRs of developmentally expressed mRNAs have been associated with regulated stability via the 3′-5′ exosome pathway following deadenylation [40]. The *cis-*acting AREs can interact with a variety of proteins to promote [58] or delay [59] ARE-mediated mRNA degradation (AMD). Recent studies and reviews have suggested that microRNAs can regulate post-transcriptional gene expression by targeting AMD as well as translation [60,61]. Association of evolutionarily conserved mi-R148/152 target sites along with ARE in the long isoform suggests a potential cooperation between microRNAs/microRNP and ARE-binding proteins (ARE-BPs) for ARE-mediated post-transcriptional regulation of MECP2 transcripts.

**Figure 7:**
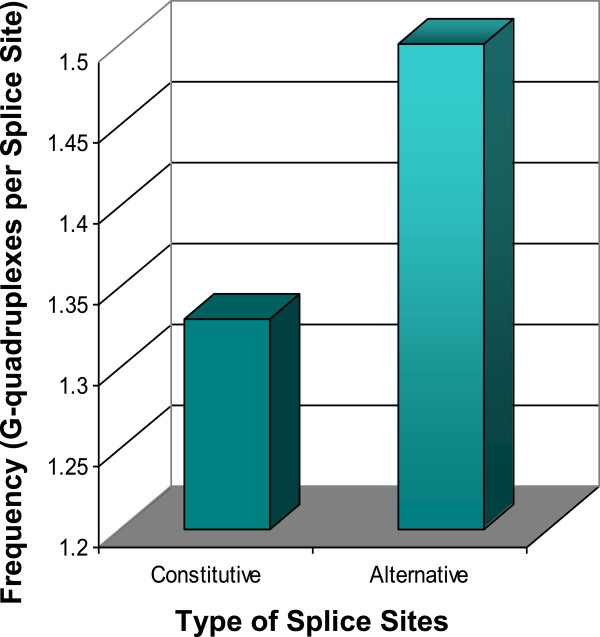
**Conserved G-quadruplexes are more likely to be associated with alternative splice sites of the mammalian MECP2 orthologs.** A total of 33 G-quadruplexes, conserved in the mammalian orthologs, were mapped to 18 constitutive and 6 alternative splice sites.

### Conserved G-quadruplex motifs near splice sites of the MECP2 pre-mRNA orthologs

We focused our attention to the conserved G-quadruplex motifs located in the vicinity of splice sites, especially those that are alternatively regulated. Human, dog, and mouse MECP2 orthologs are known to have two alternatively spliced isoforms each. The human isoform 1 (also known as MECP2A) of MECP2 has an extra exon. This isoform is predominantly expressed in the neurons during early development while the human isoform 2 is prevalent in adults in a variety of tissues including the brain.

**Figure 8:**
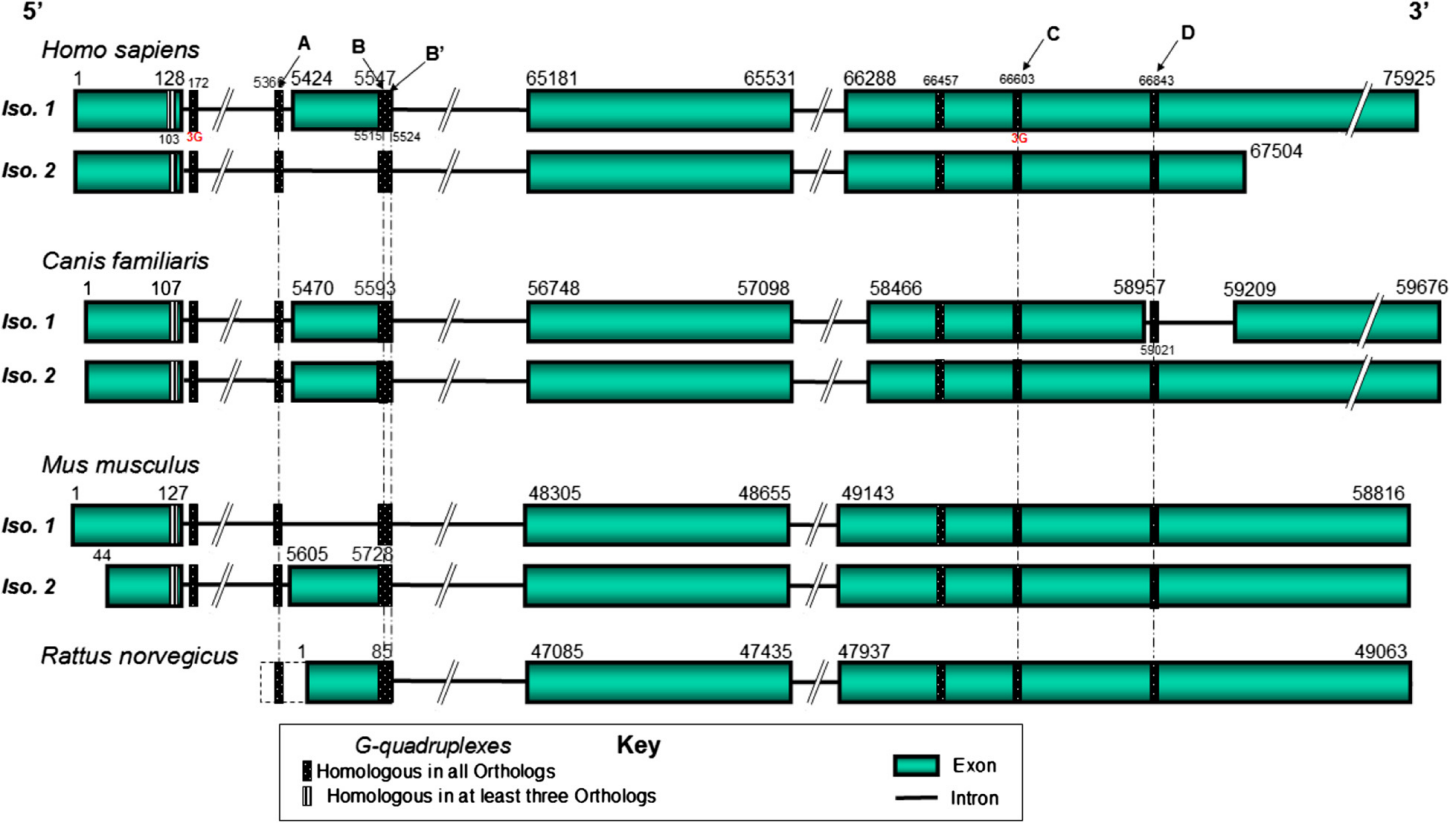
**The conserved G-quadruplex map of MECP2 pre-mRNA orthologs.** Conserved G-quadruplexes were mapped to all known alternatively spliced isoforms of MECP2 mammalian orthologs. G-quadruplex locations are highly conserved near alternative splice sites. G-quadruplexes associated with the constitutive splice sites were less likely to be conserved in their locations (data not shown). G-quadruplexes B and B′ overlap each other. The B′ G-quadruplex also overlaps the second 5′ splice site which is alternatively spliced. Four highly conserved G-quadruplexes (marked with arrows as A, B/B′, C and D) were subjected to further sequence analysis. The dotted line before the Rat MECP2 first exon represents an extension of the genomic sequence upstream to the putative transcription start site.

Many G-quadruplexes were mapped in the isoforms of four mammalian pre-mRNA orthologs. A total of 33 G-quadruplexes, which were conserved in all the four mammalian orthologs, were mapped to the vicinity of 18 constitutive and 6 alternative splice sites. A bias in the overall distribution of conserved G-quadruplexes was noticed (Figure 7). Conserved G-quadruplexes were more likely to be associated with alternative splice sites of the mammalian MECP2 orthologs, suggesting a prospective biological role for them in regulated splicing. Almost all the alternatively spliced sites of MECP2 mammalian orthologs were associated with at least one conserved G-quadruplex (Figure 8). Alternative splice site G-quadruplexes were more or less equally distributed among exons and introns.

G-quadruplex forming sequences have the potential to affect alternative tissue-specific splicing through their interactions with hnRNP H family of proteins [62]. For example, the hnRNP F protein, with an affinity for quadruplex forming G-rich sequences, is needed for nervous tissue-specific alternative splicing [10]. A G-quadruplex in FMR1 RNA can act as an alternative exonic enhancer by binding to its own FMRP protein involved in mental retardation [29]. An intronic G-quadruplex in the tumor suppressor TP53 gene is also responsible for alternative splicing [63]. A G-quadruplex in the third exon of beta-site APP cleaving enzyme 1 (BACE1) involved in Alzheimer disease has been shown to regulate splice site selection [30]. Alternative splicing in the human and mouse MECP2 pre-mRNAs involve the second exon which gets skipped. Conserved G-quadruplexes were located near both splice sites of this skippable exon in the human and mouse MECP2 orthologs. While one of the G-quadruplexes (A) was near the 3′ splice site in the intron, there were two conserved overlapping G-quadruplexes (B/B′) near the 5′ splice site in this exon. The locations of these conserved G-quadruplexes seem optimal for direct involvement in the regulated, development-related alternative splicing via interactions with splice regulatory proteins. In one of the dog MECP2 isoforms, the last exon gets interrupted by a short intron resulting in a total of five rather than four exons due to this alternative splicing (Figure 8). A conserved G-quadruplex was also discovered near the alternative 5′ splice site of the alternative intron. Our findings from this experiment suggest a good possibility that G-quadruplexes are involved in regulated alternative splicing in the MECP2 gene.

**Figure 9:**
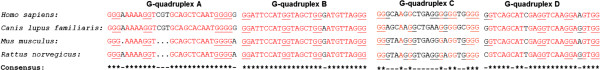
**Sequence conservation of G-quadruplex motifs in MECP2 pre-mRNA orthologs.** Location-conserved G-quadruplexes **(A**, **B**, and **D)** in the vicinity of alternative splice sites have highly conserved motifs as well. A highly stable G-quadruplex **(C)** not found near an alternate splice site is relatively less well conserved. The guanine groups which form the G-tetrads are underlined. G-quadruplexes **A**, **B**, **C**, and **D** refer to the corresponding marked map positions in Figure 8.

Multiple sequence alignments revealed that three location-conserved G-quadruplexes (A, B/B′, and D, Figure 8) near the alternative splice sites of all mammalian MECP2 orthologs have highly conserved motifs as well. A highly stable G-quadruplex (C) not found near an alternative splice site is relatively less well conserved at the sequence level (Figure 9). This data demonstrates a difference in the nature of G-quadruplexes found near alternatively spliced sites and other G-quadruplexes conserved in the same gene.

**Figure 10:**
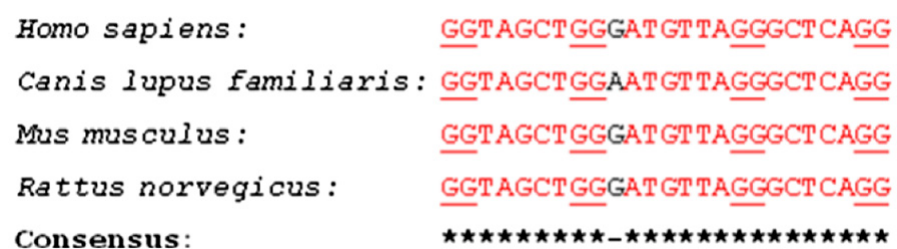
**Sequence conservation of G-quadruplex B′ motif that overlaps alternatively spliced 5′ splice site of MECP2 pre-mRNA.** Location-conserved G-quadruplex B′ (which partially overlaps with G-quadruplex B) is also highly conserved at the sequence level in all the four mammalian MECP2 orthologs. Additionally, the B′ G-quadruplex was found to overlap the second 5′ splice site of MECP2 pre-mRNA. This particular site is known to be alternatively spliced in human and mouse MECP2 orthologs. The guanine groups which form the G-tetrads are underlined. G-quadruplex B′ refers to the corresponding marked map position in Figure 8.

**Figure 11:**
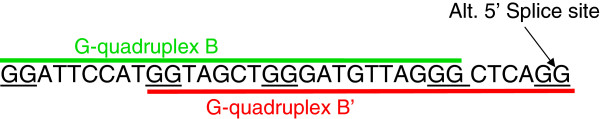
**The B/B′ G-quadruplex pair may regulate alternative splicing at the 5′ splice site of human and msouse MECP2 orthologs.** The highly conserved G-quadruplex B is found 5 bases upstream of the alternative 5′ splice site in the human MECP2 pre-mRNA sequence and may function as an exonic splicing enhancer (ESE) regulatory motif. The B′ G-quadruplex, which is also highly conserved across the mammalian species, overlaps the B motif as well as the alternative 5′ splice site. At a given time, only one of the G-quadruplex is likely to be formed in the cell. Therefore, B and B′ are likely to be mutually exclusive. G-quadruplex B′ when formed may act as an inhibitor of alternative splicing since formation of this structure is likely to make the 5′ splice site unavailable. Underlined Gs represent the bases involved in the G-tetrad formation in the G-quadruplex. G-quadruplexes B and B′ refer to the corresponding marked map positions in Figure 8. Human and mouse B/B′ G-quadruplex sequence motifs are identical.

Location-conserved G-quadruplex B′ is also highly conserved at the sequence level in all four mammalian MECP2 orthologs (Figure 10). G-quadruplex B′ partially overlaps with G-quadruplex B (Figure 8). Additionally, the B′ G-quadruplex was found to overlap the second 5′ splice site of MECP2 pre-mRNA (Figure 8). This particular site is known to be alternatively spliced in human and mouse MECP2 orthologs. The highly conserved G-quadruplex B is found 5 bases upstream of the alternative 5′ splice site in the human MECP2 pre-mRNA sequence (Figure 11). This is a convenient location for a G-quadruplex to function as an exonic splicing enhancer (ESE) regulatory motif. Previous studies have demonstrated that G-quadruplex structures found near the splice sites in the exons of genes expressed in the brain can act as ESEs by interacting with FMRP protein [29]. The B′ G-quadruplex, which is also highly conserved across the mammalian species, overlaps the B G-quadruplex motif as well as the alternative 5′ splice site. At a given time, only one of these G-quadruplexes is likely to be formed in the cell. Therefore, quadruplexes B and B′ are likely to be mutually exclusive. While G-quadruplex B can perform as an ESE, B′, when formed, may act as an inhibitor of alternative splicing since formation of this structure is likely to make the 5′ splice site unavailable. This data suggests that the B/B′ G-quadruplex pair can regulate alternative splicing in a negative as well as a positive way in the MECP2 pre-mRNAs.

Regulated alternative pre-mRNA splicing is an essential component of post-transcriptional gene expression and is important for biological processes. MECP2 produces multiple isoforms and its expression is highly regulated among different tissues, especially in the brain during different developmental stages. Our study has identified evolutionarily conserved G-quadruplexes associated with alternative splicing of MECP2 mammalian orthologs.

## Conclusions

The goal of this project was to perform evolutionary analysis of four MECP2 mammalian orthologs in order to identify conserved *cis-*regulatory elements that may regulate post-transcriptional expression of this gene which is known to be associated with mental retardation syndromes. Our bioinformatics based studies focused on G-quadruplexes, microRNA target sites, and AU-Rich elements which we mapped to the transcribed regions of MECP2 orthologs.

We identified a highly conserved G-quadruplex in the 5′-UTR of three mammalian MECP2 orthologs which overlapped with a known 11-bp deletion in Rett syndrome patients with decreased levels of MeCP2 protein but normal transcripts [42]. We believe that this 5′-UTR G-quadruplex could be involved in regulating MECP2 post-transcriptional expression either as an IRES [19,20], or by interacting with specific proteins such as nucleolin [43], or FMRP [44]. Altered levels of MeCP2 protein during the early brain development can interfere with neuronal connections, leading to autism.

The majority of the conserved *cis*-regulatory elements analyzed (G-quadruplexes, microRNA target sites, and AREs) mapped to the evolutionarily conserved regions of the otherwise variable 3′-UTR of the long MECP2 isoform which requires tight regulation during the early brain development. The short isoform which has a more stable adult expression primarily lacks most of the conserved 3′-UTR *cis-*regulatory elements analyzed. Most evolutionarily conserved G-quadruplexes were preferentially associated with microRNA target sites, suggesting an interplay between microRNAs/microRNA ribonucleoprotein (miRNP) and G-quadruplex binding proteins. A highly conserved G-quadruplex present selectively near alternative polyadenylation site 1 could be responsible for alternative polyadenylation which is the primary mechanism of differential MECP2 expression in the early brain development.

Evolutionarily conserved ARE and mi-R148/152 target sites were associated with the second alternative poly(A) site which results in the expression of longer isoform. Our data suggests that the stability and/or translation of the long MECP2 isoform, which is expected to be under strict post-transcriptional control, is potentially regulated via a cooperation between microRNAs/miRNPs and ARE-BPs.

G-quadruplex locations were found to be highly conserved near alternative splice sites of the MECP2 gene. Location-conserved G-quadruplexes in the vicinity of alternative splice sites are also highly conserved at sequence levels as compared to the G-quadruplexes found elsewhere in the MECP2 gene. We also discovered a bias in the overall distribution of conserved G-quadruplexes which were more likely to be associated with alternative splice sites of the mammalian MECP2 orthologs. Our data suggests a prospective biological role for G-quadruplexes in regulated alternative splicing of the MECP2 pre-mRNAs. We identified a pair of overlapping G-quadruplexes at an alternative 5′ splice site that could regulate alternative splicing in a negative as well as a positive way in the MECP2 pre-mRNAs.

This phylogenic analysis has provided some interesting and valuable insights into the post-transcriptional regulation of MECP2 gene by conserved *cis-*regulatory elements. The findings can help us further our understanding of mental retardation associated with this gene.

## Methods

Several freely available public databases and bioinformatics sequence analysis tools were used for this project.

### Sources of MECP2 Gene related information

The majority of the gene and sequence-related information was obtained from the database resources of National Center for Biotechnology Information (NCBI) [64]. Nucleotide and amino acid sequences of the human MECP2 gene and its orthologs were obtained from the RefSeq database [65]. The Entrez Gene database [66] was useful for obtaining alternative MECP2 isoforms and gene-related information. Exon/intron patterns were compared between the mRNA isoforms of the respective MECP2 orthologs to identify alternative and constitutive splice sites. MECP2 orthologs were identified with the help of Homologene database [64]. Several allelic variations and mutations were mapped to the human MECP2 gene with the help of OMIM database [4]. RettBASE [3] was also found to be a comprehensive collection of a wide variety of MECP2 mutations and phenotypes.

### Sequence alignments

Pairwise sequence alignments were performed with a commercial program based on the Needleman and Wunsch algorithm [67]. Unless otherwise specified, all pairwise alignments used the semi-global method rather than the full global alignment because of the variation between the lengths of untranslated regions across orthologous mRNAs. ClustalW program [68] was used for multiple sequence alignments.

### Mapping G-quadruplex sequence motifs

The QGRS Mapper [31] software program and the G-rich sequence database (GRSDB) [32] database were used to map QGRS (predicted G-quadruplexes) in the mRNA and pre-mRNA sequences of human MECP2 orthologs and generate information about the composition and distribution of QGRS in the nucleotide sequence entries. QGRS Mapper and GRSDB identify QGRS based on established algorithms which we have previously described in detail [31,69]. Briefly, the putative G-quadruplexes are identified using the motif G_x_N_y1_G_x_N_y2_G_x_N_y3_G_x_. The motif consists of four guanine (G) tracts of equal size interspersed by three loops. The size of each G-tract corresponds to the number of stacked G-tetrads forming the quadruplex structure.

While quadruplexes with at least three G-tetrads have been accepted as stable structures, two G-tetrad quadruplexes are not uncommon [70,71]. In fact, stable two G-tetrad RNA G-quadruplexes capable of significantly influencing gene expression *in vivo* have been reported [16]. Lower stability, in fact, may allow more sensitive control of gene expression [16]. Two G-tetrads are expected to be far more prevalent in the genomes as compared to the three G-tetrads. We have employed two approaches to carefully weed out potential false positive predictions. All predicted G-quadruplexes below a G-score [69] threshold of 13, representing the bottom 25% of all the G-quadruplexes in the entire human transcriptome predicted in our lab (data not presented), were discarded. Secondly, only the predicted G-quadruplexes which are phylogenetically conserved across a minimum of three mammalian MECP2 orthologs were analyzed, thereby validating our predictions.

It is widely accepted that the biological roles of G-quadruplexes depend primarily on their structure and location within the gene, rather than their sequence. The determinants of G-quadruplex homology are expected to be similarities in their specific locations on the aligned transcripts, number of tetrads, loop lengths, and overall lengths. Therefore, these criteria were adopted to identify evolutionarily conserved G-quadruplexes.

### Polyadenylation signal and site mapping

Poly(A) signals and sites information was obtained either from the NCBI nucleotide database records [65] or polyA_DB database [72] which reports evolutionarily conserved sites.

### AU-rich element mapping

AREs were mapped on the MECP2 mRNA orthologs with the help of the ARED database [73,74].

### Mapping microRNA target sites

MicroRNA target sites were mapped to the 3′-UTRs of MECP2 mRNA orthologs with the help of TargetScan [75,76] which reports target sites conserved across multiple species.

## Abbreviations

AMD: ARE-mediated mRNA degradation; APP: Amyloid precursor protein; ARE: AU-rich element; ARE-BPs: ARE-binding proteins; CDS: Coding sequence; DNA: Deoxyribose nucleic acid; ESE: Exonic splicing enhancer; FMR1: Fragile X mental retardation 1; FMRP: The fragile X mental retardation protein; GRSDB: G-rich sequence database; hnRNP: Heterogeneous nuclear ribonucleoprotein; IRES: Internal ribosomal entry site; MeCP2: Methyl CpG binding protein-2; miRNA: microRNA; miRNP: microRNA ribonucleoprotein; NCBI: National Center for Biotechnology Information; OMIM: Online mendelian inheritance in man; QGRS: Quadruplex forming G-rich sequences; RNA: Ribonucleic acid; UTR: Untranslated region; YY1: Ying Yang 1.

## Competing interests

The authors declare that they have no competing interests.

## Authors’ contributions

JB initiated the project and performed the data collection and analysis. LD helped with the design and coordination of the project and with the draft of the manuscript. Both authors have read and approved the final manuscript.

## Authors’ information

JB was a high school student when the project began. He is now studying at Carnegie-Mellon University. LD is a Professor of Mathematics and Computer Science at Ramapo College of New Jersey.
